# Real-life feasibility of home-based pulmonary rehabilitation in chemotherapy-treated patients with thoracic cancers: a pilot study

**DOI:** 10.1186/s12885-018-4102-6

**Published:** 2018-02-13

**Authors:** Cecile Olivier, Jean-Marie Grosbois, Alexis B. Cortot, Sophie Peres, Christophe Heron, Julie Delourme, Marianne Gierczynski, Anne Hoorelbeke, Arnaud Scherpereel, Olivier Le Rouzic

**Affiliations:** 1 CHU Lille, Department of Respiratory Diseases, MESOCLIN, Center for Infection and Immunity of Lille (INSERM U1019 – UMR 8204 – Pasteur Institute of Lille), University of Lille, F-59000 Lille, France; 2 Clinique de la Louvière, Pneumologie, F-59000 Lille, France; 3 FormAction Santé, F-59840 Pérenchies, France; 4 Santelys Association, F-59120 Loos, France; 5 Nutrition Department, CHU Lille, F-59000 Lille, France; 6 Department of Respiratory Diseases, CH Seclin, F-59113 Seclin, France; 7 Clinique de la Mitterie, Respiratory Diseases, F-59160 Lomme, France; 8 Pulmonary and Thoracic Oncology Department, Hôpital Calmette - CHU de Lille, F-59037 Lille Cedex, France

**Keywords:** Lung neoplasms, Mesothelioma, Pulmonary rehabilitation, Chemotherapy, Quality of life

## Abstract

**Background:**

Patients with advanced lung cancer (LC) or malignant pleural mesothelioma (MPM) exhibit limitation of exercise capacities and alteration of quality of life (QoL) induced by cancer and its treatment. Few studies assessed pulmonary rehabilitation (PR) in these chemotherapy-treated patients, and none evaluated a home-based PR program.

**Methods:**

In this prospective uncontrolled observational pilot study, patients treated by chemotherapy for LC or MPM were screened for a home-based PR program combining exercise training with global cares including therapeutic education and psychosocial management. Feasibility and safety were evaluated by attendance and adherence to PR program. Various exercise tolerance tests, including 6-min walk test (6MWT) and 6-min stepper test (6MST), were performed before and after PR associated with, QoL and psychological assessment (VSRQ and HAD, respectively).

**Results:**

243 patients were considered eligible but only 71 (60.6 ± 8.8 years) started a PR and 47 completed the program. Refusals to participate were mostly related to lack of motivation whereas withdrawals to PR were related to cancer-related medical issues. No adverse event related to PR was observed. Baseline 6MWT distance was associated with performance status (*r* = − 0.45, *p* = 0.001) and mMRC dyspnea scale (*r* = − 0.49, *p* < 0.001) but not with lung cancer stage. Post-PR reassessment showed 6MWT stability and 6MST improvement in patients who completed the program. Daily physical activity (*p* = 0.007) and anxiety (*p* = 0.02) scores were significantly improved.

**Conclusions:**

Home-based PR was feasible and safe in patients with advanced LC or MPM. Exercise capacities stability in patients who completed the PR program suggests that PR might be beneficial. Further studies are warranted to confirm and to improve the potential value of PR in these patients.

**Electronic supplementary material:**

The online version of this article (10.1186/s12885-018-4102-6) contains supplementary material, which is available to authorized users.

## Background

Lung cancer (LC) is the leading cause of death by cancer in the world since fewer than 20% of these patients could benefit from treatments with curative intent. In fact, at the time of diagnosis, most patients have advanced or metastatic stage (IIIB-IV) disease. Thus median overall survival (mOS) is still poor, closed to 12 months for stage IIIB-IV non-small cell lung cancer patients without activating mutations. The prognosis of malignant pleural mesothelioma (MPM) patients is also bleak with mOS of 13 months with standard first line treatment by cisplatin-pemetrexed chemotherapy, slightly improved up to 18.8 months in our recently published phase III trial [1, 2]. These patients with advanced thoracic cancers, often combined with several pulmonary and/or cardio-vascular comorbidities, as chronic obstructive pulmonary disease (COPD), frequently exhibit physical symptoms responsible for altered quality of life (QoL), reduced physical activity and a decline of their exercise capacities during chemotherapy [3–5]. Therefore, supportive care is essential in their treatment to counteract all these adverse effects.

Pulmonary rehabilitation (PR) is a core component of the management of individuals with chronic respiratory disease associated with improvement of symptoms, physical activity and QoL [6]. In COPD, we have previously shown that home-based PR is as safe and effective as PR performed in an outpatient rehabilitation center [7, 8]. In thoracic cancers, multiple small trials have been performed to evaluate benefits of physical activity demonstrating improvement in symptoms, exercise tolerance and QoL [9]. However, few of these studies were performed in advanced stages cancers, and none was a full home-based PR that was not only focused on exercise training.

Therefore, the aim of our study was to evaluate the feasibility of home-based PR in the real-life management of patients with unresectable LC or MPM having chemotherapy. Secondary objectives were to evaluate the safety and obtain a preliminary estimate of the effectiveness of home-based PR in these patients.

## Methods

### Study design

This prospective, observational study was conducted from March 2012 to December 2013 in the Pulmonary and Thoracic Oncology Department of the Lille University Hospital (CHU), in collaboration with the FormAction Santé, a highly trained team in PR, and supplied by Santelys, a home health care service provider. This project was approved by the observational research protocol evaluation committee of the French Language Society of Pulmonology (Comité d’Evaluation des Protocoles de Recherche Observationnels - CEPRO 2011–036).

### Patients

All consecutive patients, 18 years old or over, with confirmed histological diagnosis of LC or MPM, and treated by chemotherapy ± radiotherapy starting at the time of inclusion were screened by chest physicians and included after providing informed written consent. Exclusion criteria were usual contraindications for functional exercise testing and PR, i.e. symptomatic heart disease including unstable angina, acute pulmonary edema, acute myocarditis or pericarditis, severe cardiac rhythm disorders, musculoskeletal contraindications or other severe conditions according to the clinician advice, oncologic contraindications for PR including symptomatic bone metastasis and/or with a high risk of fracture, symptomatic brain metastasis, hemoglobin < 8 g/dL or thrombopenia < 100,000/mm^3^ and severe cognitive impairment. Patients were free to participate to the study and may refuse or stop their participations to the PR program for different reasons, which were collected, as psychological reasons (lack of motivations, more time to think needed), medical reasons (general conditions aggravation, tiredness, cancer-related issues, infection, hospital admission) or excess of constraint (patients difficulties to organize their time between cancer-related care like chemotherapy and/or radiotherapy, and PR program).

### Data collection

Patients’ assessments were performed at baseline and after 8 weeks of home-based PR. Exercise capacity was evaluated by the distance in a six-minute walk test (6MWT) and the number of steps in a six-minute stepper test (6MST) [10, 11]. The lower limb muscle strength was evaluated by the time to perform a timed Up and Go test (TUG) and a test of 10 chair stands (10CS) [12, 13]. Immediate dyspnea and lower limb muscle tiredness was quantify at the beginning and at the end of 6MWT and 6MST by a Borg scale [14]. Chronic dyspnea on exertion was quantified by the modified Medical Research Council (mMRC) scale ranging from 0 (out of breath with intensive effort) to 4 (too breathless to leave the house). QoL and psychological state of each patient were assessed using the Visual Simplified Respiratory Questionnaire (VSRQ) and the Hospital Anxiety and Depression scale (HAD) [15–17].

### Pulmonary rehabilitation (PR) program

Home-based PR was carried out for 8 consecutive weeks [8]. After performing a training diagnosis assessment, a member of the rehabilitation team (nurse, physiotherapist) provided personalized follow-up of the patient once a week at his/her home for 90 min including exercise training, resumption of daily living physical activities, therapeutic education, psychological counseling, motivational communication and nutritional advice to facilitate health behavioral changes and self-management [6]. According to this first assessment, a patient-tailored re-training program was built. Patients were educated to recognize the dyspnea threshold and were encouraged to carry out this daily exercise program independently at least 5 days per week following their personalized action plan. The re-training program lasted 30 or 45 min a day and included endurance training on cycle ergometer, muscle strengthening exercises using weights and elastic resistance band, and activity of daily living, walking and learning to climb stairs, integrated in the everyday life.

### Statistical analysis

**Fig. 1:**
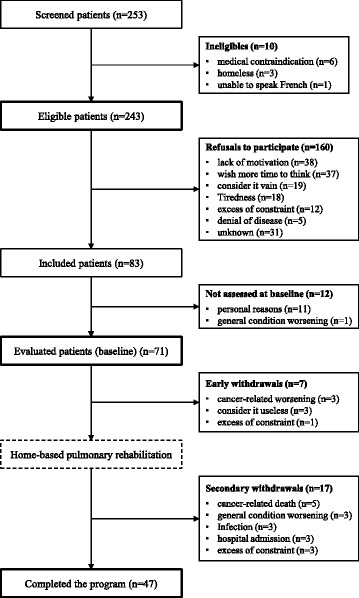
Study flowchart

Real-life feasibility of home-based PR was defined by the percentage of screened patients who completed a complete 8 weeks PR program. A description of the uptake rate defined by the percentage of screened patients who were included and of the retention rate defined by the percentage of included patients who complete the PR program was performed. This analysis was completed by description of reasons of ineligibility for this program, reasons for not participating in eligible patients and analysis of medical and individual causes of premature withdrawal of PR. Description of exercise capacity and QoL before PR relates to the 71 evaluable, enrolled patients who performed the baseline assessment (Fig. 1). Adverse events and evaluation of their link with PR program were assessed at each medical visit, i.e. once a week at home by the rehabilitation team and during each chemotherapy session by the oncology team. Comparison of matched pre- and post-PR assessment relates to the 47 patients, out of 71, who finished the PR program. This comparison was performed using a Wilcoxon test for paired data. The results are expressed as mean ± standard deviation (SD) or median with interquartile range (IQR) for quantitative variables according to distribution of data assessed with the Shapiro-Wilk test, and as numbers (%) for qualitative variables. The association between variables was tested by the Spearman rank-ordered correlation test. Differences were considered to be statistically significant when *p* ≤ 0.05.

## Results

### Attendance and adherence to home-based PR

During the recruitment period, 253 patients were screened for eligibility (Fig. 1). Only 6 patients had a medical contraindication to PR and 4 were considered ineligible for social reasons (homeless or language barrier); 65.8% of eligible patients refused to participate mostly because personal lack of motivation or time needed to think (46.9%) and few because tiredness (11.3%) or excess of constraint (7.5%). Therefore, 83 patients were included given an initial uptake of 32.8% of screened patients. After being enrolled, 11 additional patients chose to stop their participation for personal reasons and another one was excluded because of an aggravation of his medical condition. Therefore, only 71 patients have been evaluated at baseline and started the home-based PR. Most of them were men in their sixties, with smoking history and advanced lung cancer (Table 1). All of them were treated with platinum-based chemotherapy, which was their first line treatment for 52 patients out of 71 (73.2%); 6 patients were benefiting from concomitant radiotherapy, and 5 had an analgesic radiotherapy combined with chemotherapy. Finally, 24 additional patients stopped PR before the end of the program mostly for medical reasons (aggravation of their condition, infection, death) given a retention rate of 56.6% of included patients (Fig. 1). On the whole, only 18.6% of screened patients and 19.3% of eligible patients accepted to participate and finished the home-based PR.

**Table 1:** Baseline demographic and clinical characteristics of patients evaluated for pulmonary rehabilitation (*n* = 71)

Variables	
Age (mean ± SD, years)	60.6 ± 8.8
Gender (%)	
Male	76.1 (*n* = 54)
Female	23.9 (*n* = 17)
BMI (mean ± SD, kg/m^2^)	25.8 ± 8.8
Smokers (%)	
Current smokers	14.1 (*n* = 10)
Former smokers	56.3 (*n* = 40)
Non smokers	29.6 (*n* = 21)
Type of thoracic cancer (%)	
Adenocarcinoma	42.3 (*n* = 30)
Squamous cell carcinoma	16.9 (*n* = 12)
Small cell carcinoma	5.6 (*n* = 4)
Mesothelioma (MPM)	23.9 (*n* = 17)
Others	11.3 (*n* = 8)
Lung cancer TNM stage (%) excluding MPM	
stage IB	1.9 (*n* = 1)
stage IIA	5.6 (*n* = 3)
stage IIB	3.7 (*n* = 2)
stage IIIA	16.7 (*n* = 9)
stage IIIB	11.1 (*n* = 6)
stage IV	61.1 (*n* = 33)
VSRQ total score (mean ± SD)	46.3 ± 17
HAD scale	
Anxiety score (median + IQR)	7 [5–10]
Depression score (median + IQR)	6 [3–10]

For quantitative data, results are expressed as mean ± standard deviation (SD) or median with interquartile range (IQR) according to distribution of data. *SD* standard deviation, *BMI* body mass index, *MPM* malignant pleural mesothelioma *VSRQ* visual simplified respiratory questionnaire global score ranging from 80 (best health status) to 0 (poorest health status) [15], *HAD* Hospital Anxiety and Depression scale ranging from 0 (best psychological status) to 42 (worse psychological status) [17], *IQR* interquartile range

### Baseline assessment before home-based PR

At baseline, advanced lung cancer stage, WHO performance status (PS) and mMRC scores were associated with lower exercise performances (Table 2). Significant associations were also found for PS and mMRC dyspnea score but not with lung cancer stage with exercise performances (Table 3). BMI < 20 kg/m^2^ was not significantly associated with lower exercise performances (− 30 m in the 6MWT, NS) in contrast to blood albumin level < 35 g/L (− 92 m, *p* = 0.04) and prealbumin level < 0.21 g/L (− 135 m, *p* = 0.02). VSRQ and HAD total scores shown weak or no significant correlations with exercise performances (Table 3). Therefore, in our study, general condition, symptoms and albumin levels, but not disease stage, were predictive of exercise capacities. The 24 patients leaving the study after baseline evaluation were younger than the rest of the study population with more frequent stages IIIB and IV but were not significantly different in terms of PS, symptoms, exercise capacities or quality of life (Additional file 1).

**Table 2:** Exercise capacities of the patients according to their baseline characteristics (*n* = 71)

	6MWT distance (m)	6MST steps (nb)	TUG time (s)	10CS time (s)
All evaluated patients together	390 [290–450]	466 [297–572]	9 [7–14]	31 [25–47]
Lung cancer stage				
stage II	440 [395–493]	496 [412–516]	13 [11–14]	43 [27–59]
stage III	393 [323–433]	482 [372–578]	6 [5–8]	25 [25–25]
stage IV	342 [260–460]	441 [271–552]	10 [8–15]	36 [27–54]
MPM	445 [420–510]	479 [337–582]	8 [7–9]	24 [21–33]
WHO performance status				
stage 0 (*n* = 24)	430 [385–463]	516 [455–596]	6 [6–12]	23 [18–28]
stage 1 (*n* = 25)	358 [285–440]	468 [292–572]	9 [7–14]	31 [24–50]
stage 2 (*n* = 21)	290 [240–420]	282 [148–404]	9 [8–15]	36 [27–47]
mMRC dyspnea stage				
stage 0 (*n* = 4)	460 [448–473]	672 [575–676]	NA	NA
stage 1 (*n* = 26)	430 [358–470]	496 [408–579]	8 [7–10]	25 [21–29]
stage 2 (*n* = 17)	368 [250–435]	542 [466–606]	9 [6–11]	30 [19–40]
stage 3 (*n* = 9)	285 [240–290]	251 [150–276]	10 [9–14]	39 [31–48]
stage 4 (*n* = 5)	275 [170–380]	132 [71–303]	16 [11–23]	54 [38–59]

Results are given as medians with interquartile ranges. *6MWT* 6-min walk test distance, *6MST* 6-min stepper test, *TUG*: timed Up and Go test, *10CS*: ten chair stands [10–13], *MPM* malignant pleural mesothelioma, *WHO* World Health Organization, *mMRC* modified Medical Research Council dyspnea scale, *NA* not available

**Table 3:** Correlations between patients exercise capacities and their baseline characteristics (*n* = 71)

	6MWT distance (m)	6MST steps (nb)	TUG time (s)	10CS time (s)
Lung cancer stage	NS	NS	NS	NS
WHO performance status	*r* = − 0.45, *p* = 0.001	*r* = − 0.49, *p* < 0.001	NS	*r* = 0.45, *p* = 0.007
mMRC dyspnea stage	*r* = − 0.50, *p* < 0.001	*r* = − 0.50, *p* < 0.001	NS	*r* = 0.51, *p* = 0.004
VSRQ total score	*r* = 0.36, *p* = 0.018	*r* = 0.41, *p* = 0.002	NS	*r* = − 0.36, *p* = 0.03
HAD total score	NS	*r* = − 0.28, *p* = 0.04	NS	*r* = 0.35, *p* = 0.04

Correlations were tested by the spearman rank-ordered correlation test. Differences were considered to be statistically significant when *p* ≤ 0.05. *6MWT* 6-min walk test distance, *6MST* 6-min stepper test, *TUG* timed Up and Go test, *10CS* ten chair stands [10–13]. *WHO* World Health Organization, *mMRC* modified Medical Research Council dyspnea scale, *VSRQ* visual simplified respiratory questionnaire global score ranging from 80 (best health status) to 0 (poorest health status) [15], *HAD* Hospital Anxiety and Depression scale ranging from 0 (best psychological status) to 42 (worse psychological status) [17], *NS* not significant

### Evolution after home-based PR

After rehabilitation, the 6MWT distance was stable (− 5 m [− 56, 51], NS) with an increase of the 6MST number of steps (+ 63 steps [− 6, 118], *p* = 0.02), especially for MPM patients (+ 106 steps [96, 132], *p* < 0.01) (Table 4). At the end of the 6MWT, there were no significant decrease in their dyspnea score (±0 [− 2, 0], NS) and in their lower limb muscle tiredness (− 0.5 [− 2, 0], NS). However, there was a decrease time needed to achieve the 10CS (− 1.5 s [− 9, 0], *p* = 0.04) reflecting an improvement for balance and quadricipital strength but not to achieve the TUG (− 1 s [− 2, 0], *p* = 0.054). Indeed, dyspnea score was steady during follow-up (±0 [0, 0], NS) (data not shown). Although QoL improvement was not significant in all patients who completed PR program (VSRQ + 4 points [− 5, 11], *p* = 0.06), there was a significant increase of global VSRQ in MPM patients (+ 9 points [6, 14], *p* = 0.02) and of the question focusing on daily activities for all patients (*p* < 0.01) (Table 4). Finally, there was a significant decrease of the HAD anxiety score (− 1 points [− 3, 1], *p* = 0.03) without decrease of the HAD depression score (− 1 points [− 3, 1], NS) (Table 4). Beneficial effects on exercise capacities were mostly observed in PS 0–1 patients whereas PS 2 patients exhibited a decreased 6MWT distance and stable 6MST number of steps after PR (Table 5). However, the evolution of their QoL and their anxiety and depression scores did not differ from other patients. No potential adverse events related to PR activities were reported.

**Table 4:** Exercise capacities, quality of life and psychological characteristics assessed before and after pulmonary rehabilitation (*n* = 47)

	Before PR	After PR	*p*-value
6MWT distance (m)	435 [356–461]	433 [365–450]	NS
6MST steps (nb)	455 [305–574]	493 [339–609]	0.02
TUG time (s)	9 [8–13]	8.5 [7–10]	0.054
10CS time (s)	27 [24–51]	25 [20–33]	0.04
VSRQ total score	46 [37–57]	53 [39–62]	0.06
HAD total score	11 [8–17]	10 [7–17]	0.054
Anxiety score	7 [5–10]	6 [3–8]	0.03
Depression score	4 [3–9]	5 [2–9]	NS

Exercise capacities, quality of life and psychological characteristics before and after pulmonary rehabilitation (PR) from the 47 patients who completed the pulmonary rehabilitation program were compared. 6MWT: 6-min walk test distance [10], 6MST steps: number of steps in the 6-min stepper test [11], TUG: timed Up and Go test [12], 10CS: ten chair stands [13], VSRQ: visual simplified respiratory questionnaire global score ranging from 80 (best health status) to 0 (poorest health status) [15], HAD: Hospital Anxiety and Depression scale ranging from 0 (best psychological status) to 42 (worse psychological status) [17], NS: not significant. Results are given as medians with interquartile ranges. Comparisons were performed by a Wilcoxon test for paired data. Differences were considered to be statistically significant when *p* ≤ 0.05 (*p* < 0.1 are detailed)

**Table 5:** Exercise capacities, quality of life and psychological characteristics evolution according to performance status (PS) (*n* = 47)

	PS 0 *n* = 16	PS 1 *n* = 15	PS 2 *n* = 15
6MWT distance (m)	0 [−50, 43]	+ 5 [− 20, 57]	− 90 [− 185, − 39]
6MST steps (nb)	+ 74 [2, 147]	+ 96 [52, 113]	+ 8 [− 10, 36]
TUG time (s)	0 [− 1, 1]	− 2 [− 2, − 2]	− 1 [− 3, 0]
10CS time (s)	+3 [− 1, 4]	−6 [− 9, 0]	−4 [− 10, − 1]
VSRQ total score	+ 4 [− 10, 9]	+ 4 [− 5, 13]	+ 4 [2, 9]
HAD total score	0 [−5, 1]	−3 [− 7, 0]	0 [− 3, 3]
Anxiety score	−1 [− 3, 0]	−1 [− 3, 1]	0 [− 2, 2]
Depression score	0 [−2, 1]	−2 [− 3, 1]	0 [− 3, 3]

Median evolution of exercise capacities, quality of life and psychological characteristics after pulmonary rehabilitation for the 47 patients who completed the pulmonary rehabilitation program according to baseline WHO performance status. 6MWT: 6-min walk test distance [10], 6MST steps: number of steps in the 6-min stepper test [11], TUG: timed Up and Go test [12], 10CS: ten chair stands [13], VSRQ: visual simplified respiratory questionnaire global score ranging from 80 (best health status) to 0 (poorest health status) [15], HAD: Hospital Anxiety and Depression scale ranging from 0 (best psychological status) to 42 (worse psychological status) [17]. Results are given as medians with interquartile ranges

## Discussion

To our best knowledge, this is the first study testing the feasibility of home-based pulmonary rehabilitation specifically in patients with advanced LC or MPM treated by chemotherapy. This study is limited by the small group size and by its uncontrolled and observational design due to the lack of evidence in chemotherapy and/or radiotherapy treated thoracic cancers. However, the prospective design of this pilot study seemed to us the best way to do a first evaluation of feasibility and efficacy of a home-based PR in this context. Interestingly, most of these patients were eligible for rehabilitation but a lot of them refused to participate more often for lack of motivation or interest than for reported excess of constraint. Baseline exercise capacities of patients were correlated with WHO performance status, symptoms and albumin levels but not with the extension of the disease. 33.8% of patients who really started rehabilitation program did not complete the 8-week program mostly for cancer-related issues but none for potential adverse events related to PR. Finally, we globally observed a stability of patients who completed the program with significant improvement of reported daily activities and anxiety.

Attendance of patients to PR is known to be partial. For instance, in COPD 8.3% to 49.6% of referred patients do not attend PR [18, 19]. Indeed, in our study, only 28.1% of screened patients started the PR program despite few ineligible patients. Interestingly, Quist et al. and Kuehr et al. reported in a similar population of chemotherapy-treated advanced lung cancers that only 25.9% and 38.3% of eligible patients started an exercise training program, respectively [20, 21]. Conversely, Henke et al. and Cheville et al. reported better attendance of eligible patients in studies limited to exercise training (62.9% and 71%, respectively) but their methodology were different [22, 23]. In Henke et al. study, patients started training under the supervision of a physiotherapist at the time of initiation of chemotherapy and ended after completing the third cycle of chemotherapy [22]. Synchronization of training sessions and chemotherapy cycles may have helped to increase acceptability by limiting organizational constraints. On the other hand, in Cheville et al. study, patients with metastatic lung or colorectal cancer underwent home-based exercise training after a single physiotherapy visit followed by bimonthly telephone calls leaving freedom to patient to follow this program, which may have increased its adherence [23]. Interestingly, organizational difficulties as transport problems are identified as factors limiting PR attendance and some of our patients refused to participate because of constraints notably when they had concomitant radiotherapy [24]. Furthermore, significant proportion of patients refused to be enrolled due to lack of motivation or by considering PR as vain, which may be related to both patient- and physician-related factors [18, 24, 25]. Altogether, this suggests that patients with chemotherapy-treated lung cancer may associate specific psychological conditions and constraints, which may limit their enrolment in PR and lead to specific organizational and motivational care to improve it.

In contrast, adherence of our patients to PR was good as more withdrawals were secondary to cancer-related issues and few to excess of constraint. Therefore, adherence was not different of reported adherence to PR of COPD patients [18]. Moreover, none of our withdrawals was related to potential adverse event of PR confirming that home-based PR for these patients is safe, as reported in other studies [7, 20]. This is an important result as we also included patients with brain and/or bone metastasis who are sometimes excluded from other studies, suggesting that this management may also be proposed to these patients [20]. On the whole, our results suggest the feasibility and safety of PR in patients with advanced thoracic cancers including mesothelioma, which were not recruited in previous studies.

In the present work, LC or MPM patients exhibited at baseline a quite preserved exercise capacity, as reflected by 6MWT and 6MST values, compared to our COPD patients requiring oxygen and/or non-invasive ventilation included in another PR program study [7]. As COPD is a slowly evolving disease compared to thoracic cancer, we hypothesized that functional deconditioning may occur later in the course of chest malignancies after diagnosis, emphasizing the potential need to propose earlier PR in these patients and to integrate it in supportive care [26, 27]. In fact, functional capacity in advanced lung cancer is an independent predictor of survival with 13% reduced risk of death per 50 m increase in 6MWT [28]. The observation that the better predictors of poorer exercise capacities in our study are the performance status, the mMRC dyspnea stage and the VSRQ global score, emphasizes that functional capacities reflect general condition and capacities to fight the disease and support specific treatments.

Interpretation of benefits of our home-based PR program is limited by the absence of a control group. Moreover, as most withdrawals of patients were related to cancer-issues, this could have biased our study by selecting the more stable patients. Therefore, we cannot affirm that global stability of our patients in term of exercise capacities, symptoms and quality of life is secondary to PR. However, it was demonstrated that 36% of LC patients cancer reduced or stopped walking exercise over the course of 6 months [29]. Moreover, Shallwani et al. have reported a 45 m decreased of 6MWT distance after two cycles of chemotherapy whereas Temel et al. have obtained stable walk distance after an exercise training program suggesting that stability of this parameter in our study may reflect positive effects of PR [30, 31]. This hypothesis is strengthened by the observation that patients with better PS (0–1) exhibited a better exercise capacity improvement compared to patients with worse general status (PS 2). Interestingly, there were no difference in terms of quality of life and psychological characteristics evolution suggesting that even patients with poorer PS may benefit of PR that includes therapeutic education and motivational and psychosocial cares. In contrast, we were surprised to observe that patients who withdrew of PR were younger with more frequent highest lung cancer TNM stages suggesting that they may have more aggressive disease. Consequently, further research is needed to clarify if patients with rapidly evolving cancer may benefit of PR in connection with drug therapeutics and earlier supportive cares.

Finally, assessments and outcomes in our study were standard for PR programs allowing for comparison with PR programs in other medical conditions. However, as emphasized by international experts, “PR is a comprehensive intervention based on a thorough patient assessment followed by patient-tailored therapies” [6]. In addition to eliminating the constraints of travelling to a center, we believe that home-based PR also makes it possible to adapt this care more efficiently to the patient’s own environment. This might help patients to project benefits more easily into their everyday lives and keep them longer. Standard tools do not evaluate these outcomes. Therefore, it may be appropriate in future studies to include other outcomes focused on patient goals. The *patient-generated index* in which patients formulate their own responses based on their self-defined goals or expectations may be a relevant tool to achieve this evaluation [32].

## Conclusions

Home-based pulmonary rehabilitation seems feasible and safe in patients with advanced LC or MPM. Clinical benefits and physical fitness stability were observed in patients who completed the PR program, even if these results may have been partly biased by the withdrawal of the most severe patients. Thus, further research is needed to confirm these promising preliminary results, and to explore the best strategy to improve attendance to PR and its efficiency, in conjunction with active anti-tumor treatment and supportive care. Other trials focusing on patients treated by innovative treatments such as anti-tumor immunotherapy or oral targeted therapies are also warranted.

## Additional file

Additional file 1: Comparison of patients characteristics according to the completion or not of the pulmonary rehabilitation (PR) program (Table S1). (DOCX 23 kb)

## Abbreviations

10CS: 10 chair stands
6MST: 6-min stepper test
6MWT: 6-min walk test
BMI: Body mass index
COPD: Chronic obstructive pulmonary disease
HAD: Hospital Anxiety and Depression scale
IQR: Interquartile range
LC: Lung cancer
mMRC: Modified Medical Research Council dyspnea scale
mOS: Median overall survival
MPM: Malignant pleural mesothelioma
PR: Pulmonary rehabilitation
PS: WHO performance status
QoL: Quality of life
SD: Standard deviation
TUG: Timed Up and Go test
VSRQ: Visual simplified respiratory questionnaire

## References

[1] Vogelzang NJ, Rusthoven JJ, Symanowski J, Denham C, Kaukel E, Ruffie P (2003). Phase III study of Pemetrexed in combination with Cisplatin versus Cisplatin alone in patients with malignant pleural Mesothelioma. J Clin Oncol.

[2] Zalcman G, Mazieres J, Margery J, Greillier L, Audigier-Valette C, Moro-Sibilot D (2016). Bevacizumab for newly diagnosed pleural mesothelioma in the Mesothelioma Avastin Cisplatin Pemetrexed study (MAPS): a randomised, controlled, open-label, phase 3 trial. Lancet.

[3] Akin S, Can G, Aydiner A, Ozdilli K, Durna Z (2010). Quality of life, symptom experience and distress of lung cancer patients undergoing chemotherapy. Eur J Oncol Nurs.

[4] Granger CL, McDonald CF, Irving L, Clark RA, Gough K, Murnane A (2014). Low physical activity levels and functional decline in individuals with lung cancer. Lung Cancer.

[5] Kasymjanova G, Correa JA, Kreisman H, Dajczman E, Pepe C, Dobson S (2009). Prognostic value of the six-minute walk in advanced non-small cell lung cancer. J Thorac Oncol.

[6] Spruit MA, Singh SJ, Garvey C, ZuWallack R, Nici L, Rochester C (2013). An official American Thoracic Society/European Respiratory Society statement: key concepts and advances in pulmonary rehabilitation. Am J Respir Crit Care Med.

[7] Grosbois J-M, Le Rouzic O, Monge E, Bart F, Wallaert B (2013). Comparison of home-based and outpatient, hospital-based, pulmonary rehabilitation in patients with chronic respiratory diseases. Rev Pneumol Clin.

[8] Grosbois JM, Gicquello A, Langlois C, Le Rouzic O, Bart F, Wallaert B, Chenivesse C (2015). Long-term evaluation of home-based pulmonary rehabilitation in patients with COPD. Int J Chron Obstruct Pulmon Dis..

[9] Bade BC, Thomas DD, Scott JB, Silvestri GA (2015). Increasing physical activity and exercise in lung cancer: reviewing safety, benefits, and application. J Thorac Oncol.

[10] ATS Committee on Proficiency Standards for Clinical Pulmonary Function Laboratories. ATS statement: guidelines for the six-minute walk test. Am J Respir Crit Care Med. 2002;166(1):111–7. 10.1164/ajrccm.166.1.at1102 12091180

[11] Grosbois J, Riquier C, Chehere B, Coquart J, Béhal H, Bart F (2016). Six-minute stepper test: a valid clinical exercise tolerance test for COPD patients. Int. J. Chron. Obstruct. Pulmon. Dis..

[12] Bellet RN, Francis RL, Jacob JS, Healy KM, Bartlett HJ, Adams L (2013). Timed up and go tests in cardiac rehabilitation: reliability and comparison with the 6-minute walk test. J Cardiopulm Rehabil Prev.

[13] Jones SE, Kon SSC, Canavan JL, Patel MS, Clark AL, Nolan CM (2013). The five-repetition sit-to-stand test as a functional outcome measure in COPD. Thorax.

[14] Borg GA (1982). Psychophysical bases of perceived exertion. Med Sci Sports Exerc.

[15] Perez T, Arnould B, Grosbois J-M, Bosch V, Guillemin I, Bravo M-L (2009). Validity, reliability, and responsiveness of a new short visual simplified respiratory questionnaire (VSRQ) for health-related quality of life assessment in chronic obstructive pulmonary disease. Int J Chron Obstruct Pulmon Dis.

[16] Aaronson NK, Ahmedzai S, Bergman B, Bullinger M, Cull A, Duez NJ (1993). The European Organization for Research and Treatment of cancer QLQ-C30: a quality-of-life instrument for use in international clinical trials in oncology. J Natl Cancer Inst.

[17] Lepine JP, Godchau M, Brun P (1985). Anxiety and depression in inpatients. Lancet.

[18] Keating A, Lee A, Holland AE (2011). What prevents people with chronic obstructive pulmonary disease from attending pulmonary rehabilitation? A systematic review. Chron Respir Dis.

[19] Jones SE, Green SA, Clark AL, Dickson MJ, Nolan A-M, Moloney C (2014). Pulmonary rehabilitation following hospitalisation for acute exacerbation of COPD: referrals, uptake and adherence. Thorax.

[20] Quist M, Rørth M, Langer S, Jones LW, Laursen JH, Pappot H (2012). Safety and feasibility of a combined exercise intervention for inoperable lung cancer patients undergoing chemotherapy: a pilot study. Lung Cancer.

[21] Kuehr L, Wiskemann J, Abel U, Ulrich CM, Hummler S, Thomas M (2014). Exercise in patients with non-small cell lung cancer. Med Sci Sports Exerc.

[22] Henke CC, Cabri J, Fricke L, Pankow W, Kandilakis G, Feyer PC (2014). Strength and endurance training in the treatment of lung cancer patients in stages IIIA/IIIB/IV. Support. Care cancer off. J Multinatl Assoc Support Care Cancer.

[23] Cheville AL, Kollasch J, Vandenberg J, Shen T, Grothey A, Gamble G (2013). A home-based exercise program to improve function, fatigue, and sleep quality in patients with stage IV lung and colorectal cancer: a randomized controlled trial. J Pain Symptom Manag.

[24] Hayton C, Clark A, Olive S, Browne P, Galey P, Knights E (2013). Barriers to pulmonary rehabilitation: characteristics that predict patient attendance and adherence. Respir Med.

[25] Cheville AL, Rhudy L, Basford JR, Griffin JM, Flores AM (2017). How receptive are patients with late stage cancer to rehabilitation services and what are the sources of their resistance?. Arch Phys Med Rehabil.

[26] Temel JS, Greer JA, Muzikansky A, Gallagher ER, Admane S, Jackson VA (2010). Early palliative Care for Patients with metastatic non–small-cell lung cancer. N Engl J Med.

[27] Jensen W, Bialy L, Ketels G, Baumann FT, Bokemeyer C, Oechsle K (2014). Physical exercise and therapy in terminally ill cancer patients: a retrospective feasibility analysis. Support. Care cancer off. J. Multinatl. Assoc. Support. Care Cancer.

[28] Jones LW, Hornsby WE, Goetzinger A, Forbes LM, Sherrard EL, Quist M (2012). Prognostic significance of functional capacity and exercise behavior in patients with metastatic non-small cell lung cancer. Lung Cancer Amst Neth.

[29] Lin Y-Y, Liu MF, Tzeng J-I, Lin C-C (2015). Effects of walking on quality of life among lung cancer patients: a longitudinal study. Cancer Nurs.

[30] Shallwani SM, Simmonds MJ, Kasymjanova G, Spahija J (2016). Quality of life, symptom status and physical performance in patients with advanced non-small cell lung cancer undergoing chemotherapy: an exploratory analysis of secondary data. Lung Cancer.

[31] Temel JS, Greer JA, Goldberg S, Vogel PD, Sullivan M, Pirl WF (2009). A structured exercise program for patients with advanced non-small cell lung cancer. J Thorac Oncol Off Publ Int Assoc Study Lung Cancer.

[32] Tang JA, Oh T, Scheer JK, Parsa AT (2014). The current trend of administering a patient-generated index in the Oncological setting: a systematic review. Oncol Rev.

