# Supplementary material for: Real-life feasibility of home-based pulmonary rehabilitation in chemotherapy-treated patients with thoracic cancers: a pilot study
Source: BMC Cancer. 2018 Feb 13;18:178. doi: 10.1186/s12885-018-4102-6 (PMC5810120; doi:10.1186/s12885-018-4102-6)
Supplement: Supplementary file 1 — Comparison of patients characteristics according to the completion or not of the pulmonary rehabilitation (PR) program (Table S1). (DOCX 23 kb) [file 12885_2018_4102_MOESM1_ESM.docx]

**Additional file 1: Comparison of withdrawn patients to patients who completed the pulmonary rehabilitation program.**

|  | Variables | Withdrawn (n=24) | Complete (n=47) | p-value |
| --- | --- | --- | --- | --- |
|  |  |  |  |  |
|  | Age (mean ± SD, years) | 57.1 ± 10.1 | 62.4 ± 7.5 | 0.02 |
|  |  |  |  |  |
|  | Gender (%) |  |  |  |
|  | Male | 83.3 (n=20) | 72.3 (n=34) | NS |
|  | Female | 16.7 (n=4) | 27.7 (n=13) |  |
|  |  |  |  |  |
|  | BMI (mean ± SD, kg/m^2^) | 26.8 ± 13.9 | 25.3 ± 5.1 | NS |
|  |  |  |  |  |
|  | Smokers (%) |  |  |  |
|  | Current smokers | 20.8 (n=5) | 10.6 (n=5) | NS |
|  | Former smokers | 45.8 (n=11) | 61.7 (n=29) |  |
|  | Non smokers | 33.3 (n=8) | 27.7 (n=13) |  |
|  |  |  |  |  |
|  | Type of thoracic cancer (%) |  |  |  |
|  | Adenocarcinoma | 54.2 (n=13) | 36.2 (n=17) | NS |
|  | Squamous cell carcinoma | 20.8 (n=5) | 14.9 (n=7) |  |
|  | Small cell carcinoma | 4.2 (n=1) | 6.4 (n=3) |  |
|  | Mesothelioma (MPM) | 12.5 (n=3) | 29.8 (n=14) |  |
|  | Others | 8.3 (n=2) | 12.8 (n=6) |  |
|  |  |  |  |  |
|  | Lung cancer TNM stage (%) excluding MPM | |  |  |
|  | stage IB | 4.2 (n=1) | 0 (n=0) | 0.04 |
|  | stage IIA | 4.2 (n=1) | 4.3 (n=2) |  |
|  | stage IIB | 4.2 (n=1) | 2.1 (n=1) |  |
|  | stage IIIA | 4.2 (n=1) | 17.0 (n=8) |  |
|  | stage IIIB | 20.8 (n=5) | 2.1 (n=1) |  |
|  | stage IV | 50.0 (n=12) | 44.7 (n=21) |  |
|  |  |  |  |  |
|  | WHO performance status (%) |  |  |  |
|  | stage 0 | 33.3 (n=8) | 34.0 (n=16) | NS |
|  | stage 1 | 41.7 (n=10) | 31.9 (n=15) |  |
|  | stage 2 | 25.0 (n=6) | 31.9 (n=15) |  |
|  |  |  |  |  |
|  | mMRC dyspnea stage |  |  |  |
|  | stage 0 | 4.2 (n=1) | 6.4 (n=3) | NS |
|  | stage 1 | 41.7 (n=10) | 34.0 (n=16) |  |
|  | stage 2 | 25.0 (n=6) | 23.4 (n=11) |  |
|  | stage 3 | 4.2 (n=1) | 17.0 (n=8) |  |
|  | stage 4 | 8.3 (n=2) | 6.4 (n=3) |  |
|  |  |  |  |  |
|  | Exercise capacities |  |  |  |
|  | 6MWT distance (m) | 388 [275-441] | 390 [300-450] | NS |
|  | 6MST steps (nb) | 476 [305-531] | 466 [292-578] | NS |
|  | TUG time (s) | 7.5 [6-13] | 10 [8-13.5] | NS |
|  | 10CS time (s) | 31 [25.5-42] | 31.5 [25-48.5] | NS |
|  |  |  |  |  |
|  | VSRQ total score | 45 [34-53] | 46 [37-57] | NS |
|  |  |  |  |  |
|  | HAD total score | 18 [9-25] | 11 [8-18] | NS |
|  | Anxiety score | 7 [5-11] | 7 [5-10] | NS |
|  | Depression score | 9 [4-12] | 4 [3-9] | NS |
|  |  |  |  |  |

Comparison of characteristics of the 24 patients who withdrew the pulmonary rehabilitation (PR) program before the end to the 47 patients who completed the PR. SD: standard deviation, BMI: body mass index, MPM: malignant pleural mesothelioma, WHO: World Health Organization, 6MWT: 6-minute walk test, 6MST steps: number of steps in the 6-minute stepper test, TUG: timed Up and Go test, 10CS: ten chair stands, VSRQ: visual simplified respiratory questionnaire global score ranging from 80 (best health status) to 0 (poorest health status), HAD: Hospital Anxiety and Depression scale ranging from 0 (best psychological status) to 42 (worse psychological status), NS: not significant. For quantitative data, results are expressed as mean ± standard deviation (SD) or median with interquartile range (IQR) according to distribution of data. Comparisons were performed by a Student’s t-test or a Wilcoxon test for paired data according to distribution of quantitative data and a Fisher’s exact test for qualitative data. Differences were considered to be statistically significant when p ≤ 0.05.
